# Comparison of apolipoprotein B/A1 ratio, TC/HDL-C, and lipoprotein (a) for predicting outcomes after PCI

**DOI:** 10.1371/journal.pone.0254677

**Published:** 2021-07-13

**Authors:** Hae Won Jung, Seung-Pyo Hong, Kee-Sik Kim

**Affiliations:** Department of Cardiology, Daegu Catholic University Medical Center, Daegu, Korea; Harvard Medical School, UNITED STATES

## Abstract

**Background and aims:**

The Apo B/A1 ratio is a major factor that predicts future cardiovascular outcomes. However, it is unclear whether the apolipoprotein B (Apo B)/apolipoprotein A1 (Apo A1) is a better predictor of future outcome than the total cholesterol (TC)/HDL-C ratio or lipoprotein (a) (Lp (a)) after the percutaneous coronary intervention (PCI). Therefore, we performed this study to evaluate the impact of the Apo B/A1 ratio on the patients who achieved LDL-C below 70 mg/dL one year after PCI.

**Methods:**

We included 448 PCI patients whose LDL-C levels were below 70 mg/dL at follow-up. The Apo B/A1 ratio, TC/HDL-C ratio, and Lp (a) levels were measured at the time of PCI and follow-up, and decreases in these parameters between baseline and follow-up were assessed as potential markers to predict major cardiovascular adverse events (MACEs).

**Results:**

During a median follow-up period of 38.0 months, 115 MACEs were recorded. The tertile with the lowest decrease in the Apo B/A1 ratio (≤ 0.146) showed a lower MACE survival rate compared to the other tertiles. There were no differences in MACE survival rates for the TC/HDL-C ratio or Lp (a) levels.

**Conclusions:**

The Apo B/A1 ratio had better predictive accuracy for clinical outcomes compared to the TC/HDL-C ratio and Lp (a) level. A lower decrease in the Apo B/A1 ratio may be a residual risk factor for MACEs in patients who have reached LDL-C levels below 70 mg/dL after PCI.

## Introduction

The 2018 American Heart Association (AHA)/American College of Cardiology (ACC) guideline strongly suggests that low-density lipoprotein cholesterol (LDL-C) should be maintained below 70 mg/dL in patients with clinical atherosclerotic cardiovascular disease by using statins to achieve 50% or greater reduction [1]. However, the residual cardiovascular risk factor is not clear in patients whose LDL-C levels are maintained below 70 mg/dL after percutaneous coronary intervention (PCI).

The apolipoprotein B (Apo B) is the main component of very low-density lipoproteins (VLDL), intermediate-density lipoproteins (IDL), low-density lipoproteins (LDL) and lipoprotein (a) (Lp(a)); while Apolipoprotein A1 (Apo A1) is the main apolipoprotein incorporated into high‐density lipoprotein (HDL). Therefore, the Apo B/A1 ratio reflects the cholesterol balance between atherogenic and anti-atherogenic lipoprotein particles [2, 3]. Lp(a) is a low-density lipoprotein like particle with Apo B-100, linked by a disulfide bond to apolipoprotein (a). Lp(a) has enhanced atherogenic and thrombogenic properties and has been identified as an independent risk factor for cardiovascular disease [4]. Total cholesterol (TC)/high-density lipoprotein cholesterol (HDL-C) ratio has been reported as a risk indicator with greater predictive value than isolated parameters, particularly LDL [5]. In previous studies, high Apo B/A1 ratios, TC/HDL-C ratios, and Lp (a) levels have been associated with cardiovascular disease [2–4, 6–16]. However, it is unclear whether the Apo B/A1 ratio is similar to or better than the TC/HDL-C ratio or Lp (a) levels as a clinical predictor in patients who have achieved the recommended target level of LDL-C after PCI. Therefore, for the first time, we investigated the impact of Apo B/A1 ratios on clinical outcomes for patients who achieved LDL-C levels below 70 mg/dL 1 to 3 years after PCI with drug eluting stents (DES).

## Materials and methods

### Study population and data collection

**Fig 1:**
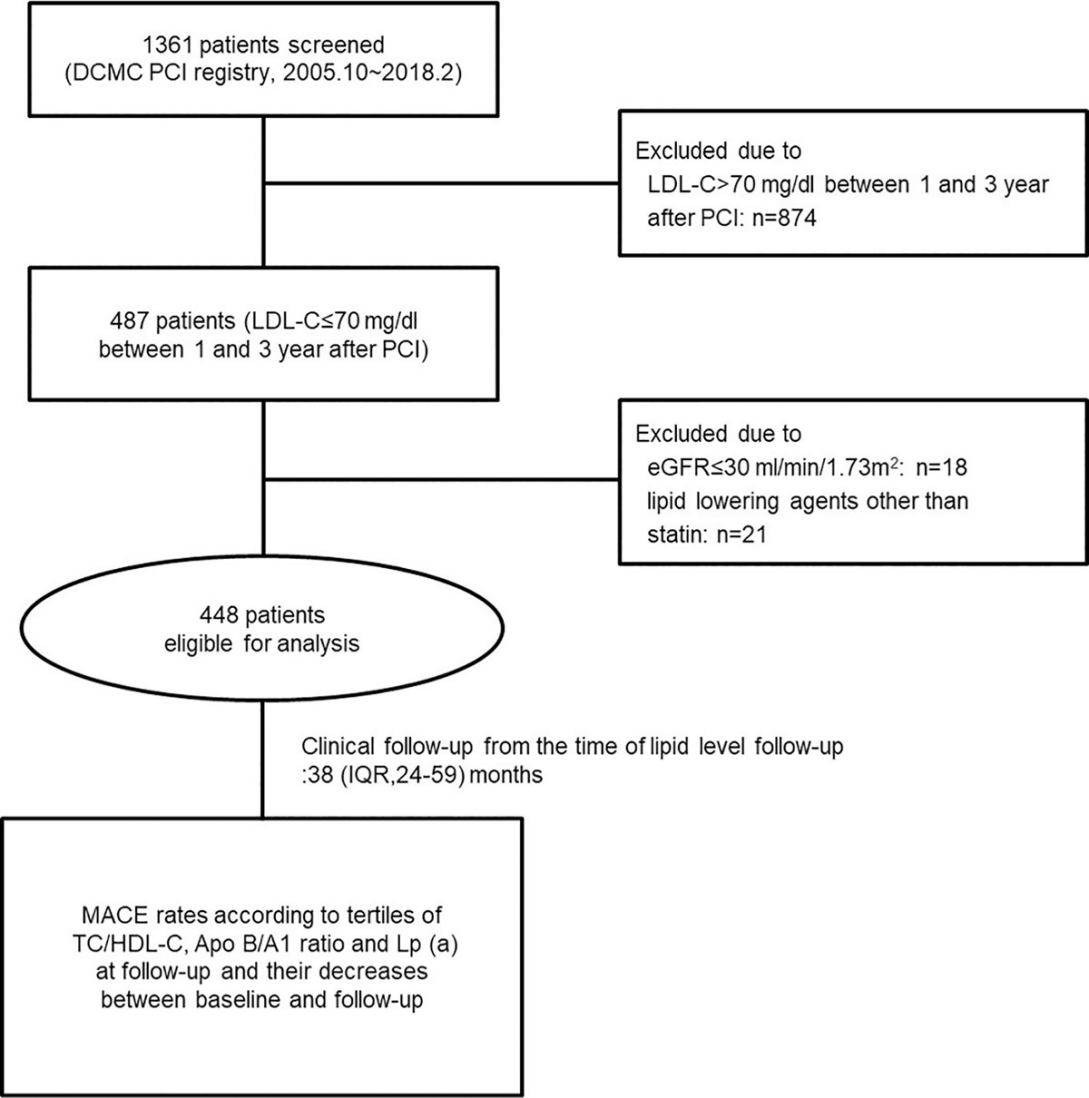
Enrollment flow chart for analysis. Apo A1; apolipoprotein A1, Apo B; apolipoprotein B, DCMC; Daegu catholic medical center, eGFR; estimated glomerular filtration rate, HDL-C; high-density lipoprotein cholesterol, LDL-C; low-density lipoprotein cholesterol, Lp (a); lipoprotein (a), MACE; major cardiovascular adverse event, PCI; percutaneous coronary intervention, TC; total cholesterol.

The study population was selected from the PCI registry at the Daegu Catholic Medical Center (Daegu, Korea). Between October 2005 and February 2018, 1,361 patients underwent their first PCI with DES and began statin therapy. Among the 1,361 patients, we investigated 487 patients whose LDL-C was below 70 mg/dL between 1 and 3 years after the PCI. Of 487 patients, we excluded 18 patients who had an estimated glomerular filtration rate below 30 mL/min/1.73 m^2^ and 21 patients who used lipid lowering agents other than statins. The inclusion and exclusion criteria for the study are shown in a flow diagram (Fig 1).

We included 448 patients in our study and evaluated the lipid profiles of these patients at the time of the PCI (baseline) and follow-up. The lipid profiles included measurements of Lp (a), TG, TC, LDL-C, HDL-C, Apo B, and Apo A1. Patients were classified into tertiles based on the Apo B/A1 and TC/HDL-C ratios and Lp (a) levels at follow-up and the differences in these parameters between baseline and follow-up. The primary endpoint was major adverse cardiovascular events (MACEs), which were defined as cardiac death, non-fatal myocardial infarction (MI), any coronary revascularization, and ischemic stroke. MI was defined by the third universal definition [17]. Clinical follow-up began at the time of the lipid profile follow-up. MACE rates were compared according to the tertile classification described above. Prescribed statins were evaluated at baseline and follow-up and classified according to statin intensity based on the ACC guideline [1]. The study protocol conformed to the ethical guidelines of the 1975 Declaration of Helsinki. The Institutional Review Board at our center approved the study and waived the requirement for patient informed consent because of the study’s retrospective nature (CR20224L).

### PCI procedure and statin usage

The type of stent selected for each patient was at the discretion of the physician at the time of the PCI. Patients were treated with first (n = 54) or second (n = 394) generation DES. First-generation DES eluted sirolimus (Cypher) or paclitaxel (Taxus), whereas second-generation DES eluted agents other than sirolimus or paclitaxel. DES implantation was performed using conventional techniques. Unfractionated heparin was administered as an initial IV bolus of 100 IU/kg body weight, with additional heparin administered during the procedure to achieve an activated clotting time of 250 to 300 s. Dual oral antiplatelet therapy (100 mg aspirin and 75 mg clopidogrel, 180 mg ticagrelor, or 10 mg prasugrel) was recommended to all patients for at least 12 months after DES implantation. Each of the 448 patients was discharged with a statin prescription after the PCI, and the type of statin selected was at the discretion of the physician.

### Statistical analysis

Data were expressed as the number (%), mean ± standard deviation, or median with interquartile range (IQR). Categorical data were compared by the chi-square test or Fisher’s exact test. Continuous variables were compared using a Student’s t-test when normally distributed and Kruskal-Wallis H test when non-normally distributed. Event-free survival was analyzed using Kaplan-Meier survival curves and compared using the log-rank test between different groups. Univariate Cox proportional hazards regression was performed to identify potential independent predictors for MACE. Age, sex and variables achieving a p-value less than 0.10 in the univariate analysis were entered in the multivariate analysis to determine the independent predictors for MACE. In the regression models, the Apo B/A1 and TC/HDL-C ratios, Lp (a) levels, and their differences between baseline and follow-up were analyzed as categorical variables and the distributions were divided into tertiles. Diagnostic accuracy of decreases in TC/HDL-C, Lp (a), Apo B/A1 ratio and LDL-C on a continuous scale in the prediction of MACE was estimated as the area under the curve (AUC) from receiver operating characteristic (ROC) curves. Univariate analysis using logistic regression was performed to identify independent predictors of the tertile showing the lowest decrease in the Apo B/A1 ratio. Age, sex and variables achieving a p-value less than 0.10 were entered in the multivariate analysis. A p-value less than 0.05 was considered statistically significant. Statistical analyses were performed using SPSS version 20.0.0 (IBM, Armonk, NY, USA).

## Results

Of the 448 patients, the mean age was 63.5 ± 10.7 years, 72.5% were men, and 49.1% were diabetic patients. After the PCI, 229 patients were discharged with a high-intensity statin dose (20 mg rosuvastatin, n = 81; 40 mg atorvastatin, n = 148) and 219 patients were discharged with a moderate intensity statin dose (5 mg rosuvastatin, n = 1; 10 mg rosuvastatin, n = 39; 10 mg atorvastatin, n = 72; 20 mg atorvastatin, n = 66; 2 mg pitavastatin, n = 36; 4 mg pitavastatin, n = 3; 20 mg simvastatin, n = 1; 40 mg pravastatin, n = 1). At the time of lipid profile follow-up, 190 patients were using a high-intensity statin dose (20 mg rosuvastatin, n = 65; 40 mg atorvastatin, n = 125) and 258 patients were taking a moderate intensity statin dose (5 mg rosuvastatin, n = 14; 10 mg rosuvastatin, n = 53; 10 mg atorvastatin, n = 76; 20 mg atorvastatin, n = 58; 2 mg pitavastatin, n = 49; 4 mg pitavastatin, n = 5; 20 mg simvastatin, n = 1; 40 mg pravastatin, n = 2). The median duration from index PCI to lipid profile follow-up was 14.0 months (interquartile range: 12.0–22.0 months). The median duration of clinical follow-up was 38.0 months (interquartile range: 24.0–59.0 months).

### Characteristics according to tertiles for the decrease in Apo B/A1 ratio

The clinical characteristics of the patients stratified by tertiles for the decrease in Apo B/A1 ratios between the PCI and lipid profile follow-up are displayed in **Table 1**. The proportion of hypertensive and male patients was highest in tertile 1 and tertile 3, respectively. At the time of both PCI and lipid profile follow-up, the incidence of high-intensity statin use was the lowest in tertile 1 and highest in tertile 3. In lipid profile evaluation, significant differences between tertiles were observed for TC, LDL-C, Apo B, HDL-C, Apo A1, and the Apo B/A1 ratio at the time of PCI. At the time of lipid profile follow-up, significant differences between tertiles were observed for HDL-C, Apo B, Apo A1, and the Apo B/A1 ratio. However, there were no significant differences for TC, TG, LDL-C, or Lp (a) among the three groups.

**Table 1:** Characteristics of individuals stratified by tertiles of Apo B/A1 ratio decrease.

Variables	Total patients (n = 448)	Tertile 1 of Apo B/A1 ratio decrease ≤0.146 (n = 149)	Tertile 2 of Apo B/A1 ratio decrease 0.146–0.346 (n = 150)	Tertile 3 of Apo B/A1 ratio decrease >0.346 (n = 149)	p-value
Clinical characteristics					
Age, years	63.5 ± 10.7	64.4 ± 10.4	63.7 ± 10.3	62.4 ± 11.3	0.238
Male	325 (72.4)	101 (67.8)	103 (68.7)	121 (81.2)	0.015
Diabetes	220 (49.0)	77 (51.7)	74 (49.3)	69 (46.3)	0.649
Hypertension	242 (53.9)	95 (63.8)	77 (51.3)	70 (47.0)	0.011
Smoking	199 (44.3)	69 (46.3)	57 (38.0)	73 (49.0)	0.137
MI at index PCI	261 (58.1)	83 (55.7)	84 (56.0)	94 (63.1)	0.343
eGFR, (ml/min/1.73 m^2^)	85.2 ± 19.1	84.8 ± 19.4	84.2 ± 19.7	86.6 ± 18.2	0.523
BMI (kg/m^2^)	23.6 ± 3.15	23.6 ± 3.57	23.7 ± 3.05	23.6 ± 2.81	0.886
EF (%)	56.1 ± 11.3	56.1 ± 11.4	56.2 ± 11.7	56.0 ± 10.8	0.989
Beta blocker	386 (86.0)	125 (83.9)	128 (85.3)	133 (89.3)	0.381
ACEi or ARB	390 (86.9)	128 (85.9)	128 (85.3)	134 (89.9)	0.435
CCB	66 (14.7)	24 (16.1)	22 (14.7)	20 (13.4)	0.807
High intensity statin at index PCI	229 (51.0)	60 (40.3)	71 (47.3)	98 (65.8)	<0.001
High intensity statin at lipid follow-up	190 (42.4)	53 (35.6)	57 (38.0)	80 (53.7)	0.003
Procedural characteristics					
Multi vessel disease	218 (48.6)	77 (51.7)	71 (47.3)	70 (47.0)	0.665
Type b2/c lesions	230 (51.2)	71 (47.7)	86 (57.3)	73 (49.0)	0.192
PCI on LAD lesion	301 (67.0)	92 (61.7)	109 (72.7)	100 (67.1)	0.132
Lipid profile at index PCI					
TC (mg/dL)	169.8 ± 35.9	151.4 ± 32.4	171.1 ± 33.6	186.8 ± 32.9	<0.001
TG (mg/dL)	157.0 ± 117.1	153.4 ± 125.8	159.3 ± 124.0	158.4 ± 100.7	0.898
HDL-C (mg/dL)	43.9 ± 12.1	47.1 ± 14.3	44.9 ± 11.5	39.6 ± 8.75	<0.001
LDL-C (mg/dL)	103.0 ± 31.9	82.0 ± 24.1	102.5 ± 28.5	124.6 ± 27.7	<0.001
Lp(a) (mg/dL)	25.5 ± 26.7	27.1 ± 28.9	24.6 ± 26.0	25.0 ± 25.3	0.681
Apo B (mg/dL)	88.2 ± 23.6	70.9 ± 17.8	85.7 ± 17.4	107.9 ± 19.0	<0.001
Apo A1 (mg/dL)	124.1 ± 26.5	132.9 ± 28.0	127.7 ± 26.4	111.8 ± 19.5	<0.001
Apo B/A1 ratio	0.740 ± 0.249	0.549 ± 0.155	0.685 ± 0.138	0.985 ± 0.209	<0.001
Lipid profile at follow-up					
TC (mg/dL)	113.5 ± 18.0	115.0 ± 19.6	114.0 ± 18.7	111.5 ± 15.4	0.231
TG (mg/dL)	113.6 ± 73.4	123.3 ± 86.3	110.6 ± 68.3	107.1 ± 63.1	0.134
HDL-C (mg/dL)	44.7 ± 13.1	42.6 ± 14.7	46.5 ± 13.0	44.9 ± 11.1	0.036
LDL-C (mg/dL)	56.0 ± 10.6	57.1 ± 9.5	55.6 ± 11.3	55.4 ± 10.9	0.295
Lp(a) (mg/dL)	25.7 ± 26.8	28.0 ± 26.8	25.2 ± 26.5	23.9 ± 27.2	0.406
Apo B (mg/dL)	57.9 ± 12.2	60.6 ± 12.6	56.3 ± 12.8	56.8 ± 10.7	0.003
Apo A1 (mg/dL)	125.8 ± 27.0	120.4 ± 29.6	130.9 ± 25.6	125.9 ± 24.6	0.003
Apo B/A1 ratio	0.485 ± 0.166	0.543 ± 0.217	0.447 ± 0.135	0.465 ± 0.113	<0.001

Data are given as mean ± SD, or as number (%). ACEi; angiotensin converting enzyme inhibitor, Apo A1; apolipoprotein A1, Apo B; apolipoprotein B, ARB; angiotensin receptor blocker, BMI; body mass index, CCB; calcium channel blocker, DES; drug-eluting stent, EF; ejection fraction, eGFR; estimated glomerular filtration rate, HDL-C; high-density lipoprotein cholesterol, LAD; left anterior descending artery, LDL-C; low-density lipoprotein cholesterol, Lp(a); lipoprotein (a), PCI; percutaneous coronary intervention, TC; total cholesterol, TG; triglyceride.

### The lipoprotein ratios and cardiovascular events

**Fig 2:**
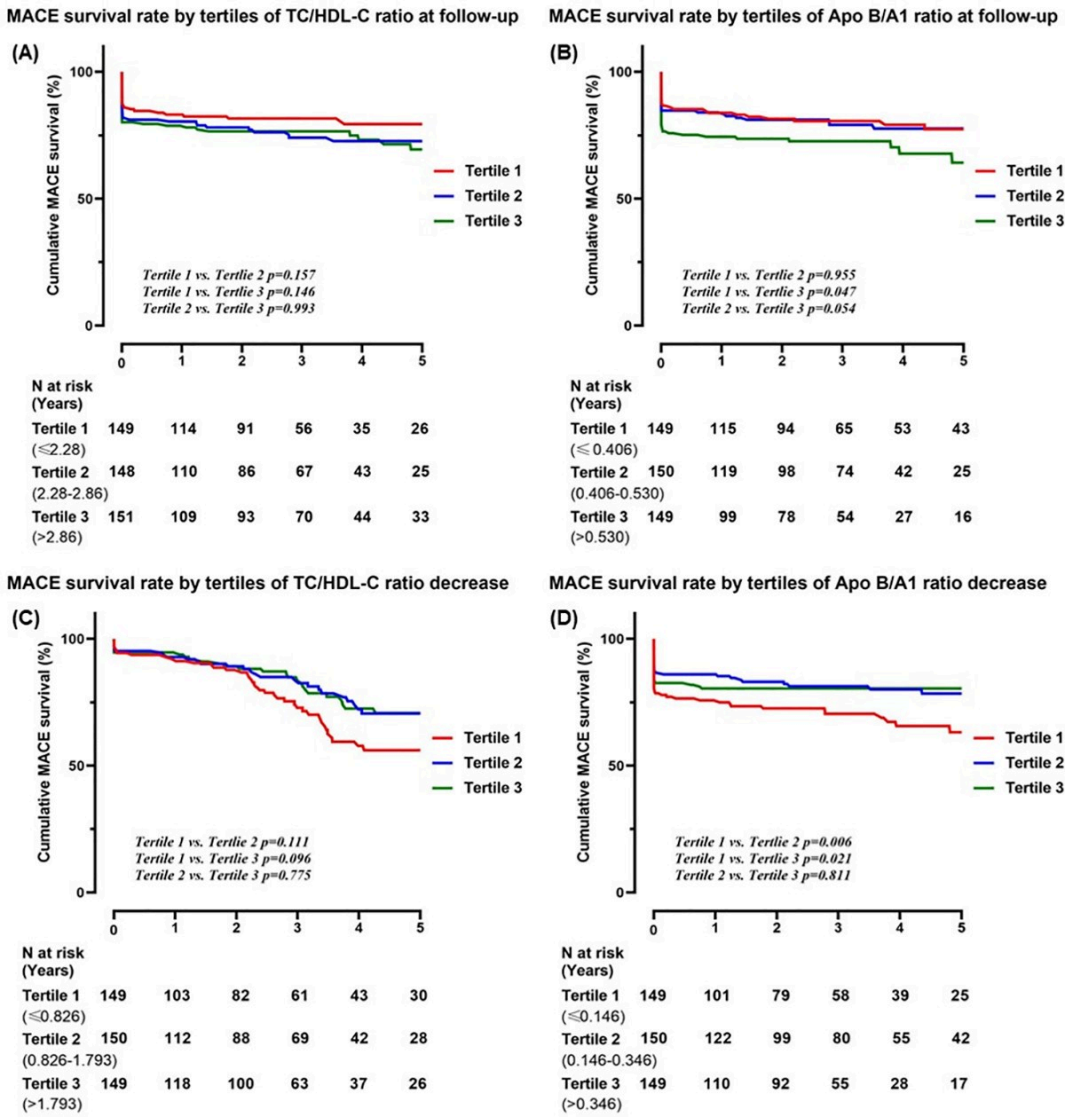
Kaplan-Meier curves of MACE survival rate by tertile of TC/HDL-C ratio at follow-up (A). Kaplan-Meier curves of MACE survival rate by tertile of Apo B/A1 ratio at follow-up (B). Kaplan-Meier curves of MACE survival rate by tertile of TC/HDL-C ratio decrease (C). Kaplan-Meier curves of MACE survival rate by tertile of Apo B/A1 ratio decrease (D). Apo A1; apolipoprotein A1, Apo B; apolipoprotein B, HDL-C; high-density lipoprotein cholesterol, MACE; major cardiovascular adverse event, TC; total cholesterol.

During the clinical follow-up, MACEs occurred in 115 patients. Kaplan-Meier curves of MACE survival rates by tertiles for the TC/HDL-C and Apo B/A1 ratios at lipid profile follow-up are summarized in **Fig 2A** and **2B**, respectively. There were no significant differences in MACE survival rates between the tertiles for the TC/HDL-C ratio at lipid profile follow-up (**Fig 2A**). However, for the Apo B/A1 ratio at lipid profile follow-up, tertile 3 showed a significantly lower MACE survival rate compared to tertile 1 and tended to have a lower MACE survival rate compared to tertile 2 (**Fig 2B**). Kaplan-Meier curves of MACE survival rates by tertiles for decreases in TC/HDL-C and Apo B/A1 ratios that were observed between PCI and follow-up are summarized in **Fig 2C** and **2D**, respectively. There were no significant differences in MACE survival rates between tertiles for the TC/HDL-C ratio decrease (**Fig 2C**). However, for the decrease in Apo B/A1 ratio, tertile 1 showed significantly lower MACE survival rate compared to tertile 2 and 3 (**Fig 2D**).

**Fig 3:**
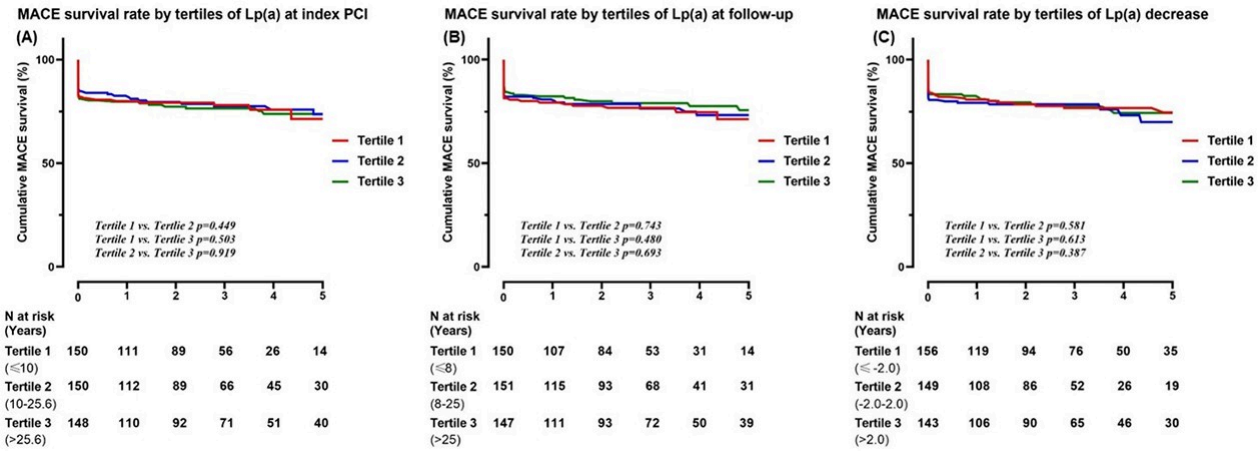
Kaplan-Meier curves of MACE survival rate by tertile of Lp (a) at index PCI (A). Kaplan-Meier curves of MACE survival rate by tertile of Lp (a) at follow-up (B). Kaplan-Meier curves of MACE survival rate by tertile of Lp (a) decrease (C). Lp (a); lipoprotein (a), MACE; major cardiovascular adverse event, PCI; percutaneous coronary intervention.

Kaplan-Meier curves of MACE survival rates by tertiles for Lp (a) at PCI and follow-up are summarized in **Fig 3A** and **3B**, respectively. Kaplan-Meier curves of MACE survival rate by tertiles for decreases in Lp (a) levels between PCI and follow-up are summarized in **Fig 3C**. There were no significant differences in MACE survival rate among tertiles for Lp (a) levels at the time of PCI, follow-up, or for decreased Lp (a) levels between PCI and follow-up.

The rates for individual MACE are summarized in **Table 2** according to tertiles based on the decrease in the Apo B/A1 ratios between the PCI and follow-up. The overall MACE, any revascularization, and cardiac death rates were significantly higher in tertile 1 than in tertile 2 and 3. The rates for individual MACE according to TC/HDL-C ratio, Apo B/A1 ratio, Lp(a), and their decreases are summarized in **S1–S3 Tables**, respectively. We also performed analysis to evaluate whether or not the achievement of LDL lowering of more than 50% from baseline was associated with improved outcomes. As a result, the group in which LDL-C decreased by more than 50% showed a significantly lower incidence of MACE compared to the group in which LDL-C decreased by less than 50% (MACE rate: 21.2% vs 30.2%, p = 0.012). The rates for individual MACE according to % reduction of LDL-C is summarized in **S4 Table**.

**Table 2:** Clinical adverse events stratified by tertiles of Apo B/A1 ratio decrease.

Variables	Tertile of Apo B/A1 ratio decrease			p-value
	Tertile 1 ≤0.146 (n = 149)	Tertile 2 0.146–0.346 (n = 150)	Tertile 3 >0.346 (n = 149)	
MACE	51 (34.2)	33 (22.0)	31 (20.8)	0.010
Any revascularization	47 (31.5)	33 (22.0)	30 (20.1)	0.039
Nonfatal-MI	5 (3.4)	4 (2.7)	5 (3.4)	0.854
Ischemic stroke	7 (4.7)	3 (2.0)	1 (0.7)	0.132
Cardiac death	5 (3.4)	1 (0)	1 (0.7)	0.033

Data are given as number (%) Apo A1; apolipoprotein A1, Apo B; apolipoprotein B, MACE; major cardiovascular adverse event (cardiac death, non-fatal myocardial infarction, any coronary revascularization and ischemic stroke), MI; myocardial infarction.

### Independent predictors of MACEs and lower decrease in the Apo B/A1 ratio

According to multivariate analysis using Cox proportional hazards regression, the rate of MACE was independently increased by multi-vessel disease at the time of PCI and the lowest tertile for the decrease in the Apo B/A1 ratio (**Table 3**).

**Table 3:** Independent predictors for MACEs.

Variable	Univariate analysis			Multivariate analysis		
	HR	95% CI	p-value	HR	95% CI	p-value
Age	1.007	0.989–1.025	0.435	1.005	0.986–1.025	0.622
Male	0.961	0.641–1.440	0.846	1.020	0.655–1.587	0.932
Diabetes	0.917	0.636–1.323	0.644			
Hypertension	1.059	0.732–1.531	0.762			
Smoking hx	0.959	0.664–1.386	0.825			
BMI	1.021	0.964–1.081	0.473			
Ejection fraction	0.993	0.978–1.009	0.416			
Multi vessel disease at index PCI	1.583	1.092–2.295	0.015	1.529	1.053–2.221	0.026
Type b2/c lesion at index PCI	0.959	0.665–1.383	0.823			
Highest tertile of Apo B/ A1 ratio at lipid follow-up	1.513	1.035–2.210	0.032	1.107	0.752–1.630	0.607
Highest tertile of Lp (a) at lipid follow-up	0.889	0.601–1.316	0.557			
Highest tertile of TC/HDL-C ratio at lipid follow-up	1.168	0.801–1.702	0.421			
Lowest tertile of Apo B/A1 ratio decrease	1.708	1.181–2.469	0.004	1.644	1.004–2.689	0.048
Lowest tertile of Lp (a) decrease	0.996	0.680–1.459	0.983			
Lowest tertile of TC/HDL-C ratio decrease	1.430	0.986–1.430	0.059	1.049	0.642–1.712	0.850
Non-high intensity statin at index PCI	0.933	0.632–1.378	0.729			
Non-high intensity statin at lipid follow-up	0.721	0.490–1.062	0.098	0.731	0.485–1.101	0.134
ACE inhibitor/ARB	0.773	0.467–1.278	0.315			
Beta blocker	0.742	0.462–1.193	0.218			
CCB	1.409	0.885–2.244	0.148			
First-generation DES	0.597	0.323–1.102	0.099	0.671	0.354–1.274	0.223

ACE; angiotensin-converting enzyme, Apo A1; apolipoprotein A1, Apo B; apolipoprotein B, ARB; angiotensin receptor antagonist, BMI; body mass index, CCB; calcium channel blocker, CI; confidence interval, DES; drug-eluting stent, HR; hazard ratio, Lp (a); lipoprotein (a), MACEs; major cardiovascular adverse events, PCI; percutaneous coronary intervention.

According to the ROC analyses, the best predictive value for MACEs was Apo B/A1 ratio decrease ≤0.1633 ([AUC] = 0.575 p = 0.017, sensitivity 46.96%, specificity 69.37%). The AUC for Apo B/A1 ratio decrease was larger than that of TC/HDL-C ratio decrease, Lp (a) decrease and LDL-C decrease. (TC/HDL-C decrease: [AUC] = 0.560, p = 0.056, Lp (a) decrease: [AUC] = 0.485, p = 0.620, LDL-C decrease: [AUC] = 0.556, p = 0.072) In multivariate analysis using logistic regression, the lowest tertile of LDL-C (≤ 87.6 mg/dL) and use of non-high-intensity statins at the time of PCI were the independent predictors of the lowest tertile for the decrease in the Apo B/A1 ratio (**Table 4**).

**Table 4:** Independent predictors for lowest tertile of Apo B/A1 ratio decrease.

Variable	Univariate analysis			Multivariate analysis		
	OR	95% CI	p-value	OR	95% CI	p-value
Age	1.013	0.994–1.032	0.183	0.988	0.966–1.012	0.328
Male	0.705	0.457–1.085	0.112	0.676	0.395–1.157	0.153
Diabetes	1.167	0.787–1.729	0.442			
Hypertension	1.819	1.125–2.724	0.004	1.531	0.959–2.444	0.075
Smoking hx	1.121	0.755–1.664	0.570			
BMI	0.987	0.927–1.051	0.692			
Lowest tertile of LDL-C at index PCI	7.982	5.113–12.461	<0.001	9.132	5.659–14.738	<0.001
Non-high intensity statin at index PCI	1.928	1.293–2.875	0.001	2.458	1.195–5.056	0.015
Non-high intensity statin at lipid profile follow up	1.532	1.021–2.297	0.039	0.928	0.446–1.929	0.840
ACE inhibitor/ARB	0.861	0.484–1.531	0.610			
Beta blocker	0.758	0.436–1.319	0.327			
CCB	1.175	0.681–2.026	0.562			

ACE; angiotensin-converting enzyme, Apo A1; apolipoprotein A1, Apo B; apolipoprotein B, ARB; angiotensin receptor antagonist, BMI; body mass index, CCB; calcium channel blocker, CI; confidence interval, LDL-C; low-density lipoprotein cholesterol, OR; odds ratio, PCI; percutaneous coronary intervention.

## Discussion

The Apo B/A1 ratio is an indicator of the balance between atherogenic and atheroprotective cholesterol transport. High Apo B/A1 ratios have been associated with poor cardiovascular outcomes, including cardiac death, MI, and ischemic stroke [2, 6–12]. The Apo B/A1 ratio has been shown to predict cardiovascular risk more accurately than the TC/HDL-C ratio [2, 6]. The primary findings of our study are as follows: 1) the Apo B/A1 ratio demonstrated a greater predictive accuracy for clinical outcomes compared with the TC/HDL-C ratio and Lp (a) levels in patients who reached the target LDL-C level of ≤ 70 mg/dL 1 to 3 years after PCI. Particularly, we showed for the first time that a decrease in the Apo B/A1 ratio over time was a better clinical predictor than the absolute value of the Apo B/A1 ratio at follow-up in patients who achieved the target level of LDL-C after PCI. 2) The risk factors that predicted the lowest decrease in the Apo B/A1 ratio were low LDL-C levels and use of non-high-intensity statins after the PCI.

Previous studies have reported that high Lp (a) levels may be associated with a poor prognosis after PCI [4, 15, 16]. Contrary to previous studies, in our study, neither Lp (a) at PCI, Lp (a) at lipid follow-up, and Lp (a) decrease were suitable for predicting future clinical outcomes after PCI. Unlike our research, previous studies did not evaluate patients whose LDL-C levels fell below 70 mg/dL after PCI and statin use. In the present study, the mean LDL-C level of lipid profile follow-up was extremely low, 56.0 ± 10.6 mg/dL. Schmidt et al. [12] have reported that for subjects in the lowest LDL tertile, the risk of having a plaque in the femoral artery was three times greater for subjects in the highest Apo B/A1 tertile compared to subjects in the lowest Apo B/A1 tertile. Given these results, it seems that the Apo B/A1 ratio has a better prognosis prediction than Lp(a) for patients who have succeeded in lowering LDL-C level to below 70 mg/dL after PCI.

The results of a meta-analysis demonstrated that the percentage reduction of LDL-C is more important for secondary prevention than for the achievement of a target LDL-C level [18]. European guidelines have suggested lowering the LDL-C level to a specific target while achieving a ≥ 50% reduction in LDL-C for very high-risk patients [19]. The idea is to avoid insufficient lipid-lowering therapy in patients with atherosclerotic cardiovascular disease and a low baseline LDL-C level. In these patients, lowering the LDL-C level to <70 mg/dL (but without a ≥ 50% reduction) using low- or moderate-intensity statins may deny them the potential outcome benefit that could be obtained if they were treated with high-intensity statins [20]. In our study, which was conducted on patients whose LDL-C remained low below 70 after PCI, the LDL-C reduction group of 50% or more showed significantly better results than the LDL-C reduction group of 50% or less. These results emphasize the use of high-intensity statins regardless of baseline LDL-C level in patients undergoing PCI. We also demonstrated that decrease in the Apo B/A1 ratio over time was a better clinical predictor than the absolute value of the Apo B/A1 ratio. In our study, the AUC for Apo B/A1 ratio decrease was larger than that of LDL-C decrease. This observation indicates that the Apo B/A1 ratio decrease may give additional information in predicting cardiovascular risk, beyond that of LDL-cholesterol decrease.

Cho et al. [21] demonstrated that patients with low LDL-C level (< 70 mg/dL) at the time of acute MI showed the poorest clinical outcomes, which may be attributable to less statin use. Lee et al [22] also investigated the clinical outcomes of statin therapy in patients with acute MI whose LDL-C levels were <70mg/dl, and found that statin therapy was associated with improved clinical outcome compared to no statin therapy. However, the authors did not suggest an underlying mechanism for linking low LDL levels and less or no statin use to poor outcome. The present study demonstrated the independent predictors of the lowest decrease in Apo B/A1 ratios were the lowest LDL-C (≤ 87.6 mg/dL) and use of non-high-intensity statin after PCI, and a lower decrease in Apo B/A1 ratio led to poor clinical outcomes.

It has been reported that the decrease in the Apo B/A1 ratio varies depending on the type and intensity of statin or addition of ezetimibe. Patients who used high-intensity statins or added ezetimibe had a more reduced Apo B/A1 ratio than those who used non-high-intensity statins [23, 24]. Tani et al. [25] demonstrated that the decrease in the Apo B/A1 ratio in response to statin therapy is a simple predictor of coronary atherosclerotic regression. If the decrease of the Apo B/A1 ratio is not sufficient even when LDL-C reaches the target level, high intensity or combination of ezetimibe should be considered for the coronary plaque regression. Currently, target lipid levels are set mainly for LDL-C to improve outcomes after PCI; however, there are no clear target levels for other lipids, especially in patients who met the current LDL-C target level after PCI [1]. Although the best predictive value for MACEs was Apo B/A1 ratio decrease ≤0.1633 in our study, large-scale studies on target Apo B/A1 ratio decrease level should be conducted in the future. The results of our study suggest the need to pay close attention to the reduction of the Apo B/A1 ratio, even if the LDL-C criteria are met after PCI, and stricter lipid profile criteria should be applied to patients with low LDL-C levels at the time of the first PCI than patients with high LDL-C levels.

Our study had several limitations. First, because of the retrospective nature of this study, there were differences in duration for lipid and clinical follow-up for each patient. However, this study was conducted with patients who had good treatment compliance and arrived at the target LDL-C 1 to 3 year after statin use, and the clinical follow-up duration (median 38.0 months) was long enough to assess the effects of the decrease in the Apo B/A1 ratio. Second, this study was a single-center study and was conducted with a relatively small number of patients. Third, since the type of statins could be changed during clinical follow-up at the discretion of the physician, it was difficult to evaluate the effects of these statin changes on the lipid profiles and future clinical outcomes.

## Conclusions

The Apo B/A1 ratio had greater predictive accuracy for clinical outcomes compared with the TC/HDL-C ratio and Lp (a) after PCI. A lower decrease in the Apo B/A1 ratio over time can be a residual risk factor of MACE in patients who have reached LDL-C levels below 70 mg/dL after PCI.

## Supporting information

S1 Table Clinical adverse events stratified by tertiles of TC/HDL-C ratio and TC/HDL-C ratio decrease. (DOCX)

S2 Table Clinical adverse events stratified by tertiles of Apo B/A1 ratio and Apo B/A1 ratio decrease. (DOCX)

S3 Table Clinical adverse events stratified by tertiles of Lp(a) and Lp(a) decrease. (DOCX)

S4 Table Clinical adverse events stratified by % reduction of LDL-C. (DOCX)
