# Supplementary material for: Comparison of apolipoprotein B/A1 ratio, TC/HDL-C, and lipoprotein (a) for predicting outcomes after PCI
Source: PLoS One. 2021 Jul 13;16(7):e0254677. doi: 10.1371/journal.pone.0254677 (PMC8277048; doi:10.1371/journal.pone.0254677)
Supplement: S1 Table — (DOCX) [file pone.0254677.s001.docx]

S1 Table. Clinical adverse events stratified by tertiles of TC/HDL-C ratio and TC/HDL-C ratio decrease.

| Variables | Tertile of TC/HDL-C ratio at lipid follow-up | | | p-value |
| --- | --- | --- | --- | --- |
|  | Tertile 1  ≤2.28 (n=149) | Tertile 2  2.28–2.86 (n=148) | Tertile 3  >2.86 (n=151) |  |
| MACE | 30 (20.1) | 41 (27.7) | 44 (29.1) | 0.279 |
| Any revascularization | 30 (20.1) | 39 (26.4) | 41 (27.2) | 0.418 |
| Nonfatal-MI | 3 (2.0) | 5 (3.4) | 6 (4.0) | 0.701 |
| Ischemic stroke | 2 (1.3) | 4 (2.7) | 5 (3.3) | 0.807 |
| Cardiac death | 2 (1.3) | 1 (0.7) | 3 (2.0) | 0.652 |
| Variables | Tertile of TC/HDL-C ratio decrease | | | p-value |
|  | Tertile 1  ≤0.826 (n=149) | Tertile 2  0.826–1.793 (n=150) | Tertile 3  >1.793 (n=149) |  |
| MACE | 48 (32.2) | 35 (23.3) | 32 (21.5) | 0.136 |
| Any revascularization | 45 (30.2) | 34 (22.7) | 31 (20.8) | 0.202 |
| Nonfatal-MI | 6 (4.0) | 3 (2.0) | 5 (3.4) | 0.600 |
| Ischemic stroke | 8 (5.4) | 3 (2.0) | 0 (0) | 0.021 |
| Cardiac death | 3 (2.0) | 3 (2.0) | 0 (0) | 0.232 |

Data are given as number (%) HDL-C; high-density lipoprotein cholesterol, MACE; major cardiovascular adverse event (cardiac death, non-fatal myocardial infarction, any coronary revascularization and ischemic stroke), MI; myocardial infarction, TC; total cholesterol.
