# Supplementary material for: Comparison of apolipoprotein B/A1 ratio, TC/HDL-C, and lipoprotein (a) for predicting outcomes after PCI
Source: PLoS One. 2021 Jul 13;16(7):e0254677. doi: 10.1371/journal.pone.0254677 (PMC8277048; doi:10.1371/journal.pone.0254677)
Supplement: S2 Table — (DOCX) [file pone.0254677.s002.docx]

S2 Table. Clinical adverse events stratified by tertiles of Apo B/A1 ratio and Apo B/A1 ratio decrease.

| Variables | Tertile of Apo B/A1 ratio at lipid follow-up | | | p-value |
| --- | --- | --- | --- | --- |
|  | Tertile 1  ≤0.406 (n=149) | Tertile 2  0.406–0.530 (n=150) | Tertile 3  >0.530 (n=149) |  |
| MACE | 37 (24.8) | 33 (22.0) | 45 (30.2) | 0.077 |
| Any revascularization | 36 (24.2) | 33 (22.0) | 41 (27.5) | 0.269 |
| Nonfatal-MI | 6 (4.0) | 3 (2.0) | 5 (3.4) | 0.647 |
| Ischemic stroke | 5 (3.4) | 3 (2.0) | 3 (2.0) | 0.959 |
| Cardiac death | 2 (1.3) | 2 (1.3) | 2 (1.3) | 0.971 |
| Variables | Tertile of Apo B/A1 ratio decrease | | | p-value |
|  | Tertile 1  ≤0.146 (n=149) | Tertile 2  0.146–0.346 (n=150) | Tertile 3  >0.346 (n=149) |  |
| MACE | 51 (34.2) | 33 (22.0) | 31 (20.8) | 0.010 |
| Any revascularization | 47 (31.5) | 33 (22.0) | 30 (20.1) | 0.039 |
| Nonfatal-MI | 5 (3.4) | 4 (2.7) | 5 (3.4) | 0.854 |
| Ischemic stroke | 7 (4.7) | 3 (2.0) | 1 (0.7) | 0.132 |
| Cardiac death | 5 (3.4) | 1 (0) | 1 (0.7) | 0.033 |

Data are given as number (%) Apo A1; apolipoprotein A1, Apo B; apolipoprotein B, MACE; major cardiovascular adverse event (cardiac death, non-fatal myocardial infarction, any coronary revascularization and ischemic stroke), MI; myocardial infarction
