# Supplementary material for: Comparison of apolipoprotein B/A1 ratio, TC/HDL-C, and lipoprotein (a) for predicting outcomes after PCI
Source: PLoS One. 2021 Jul 13;16(7):e0254677. doi: 10.1371/journal.pone.0254677 (PMC8277048; doi:10.1371/journal.pone.0254677)
Supplement: S3 Table — (DOCX) [file pone.0254677.s003.docx]

S3 Table. Clinical adverse events stratified by tertiles of Lp(a) and Lp(a) decrease.

| Variables | Tertile of Lp(a) at lipid follow-up | | | p-value |
| --- | --- | --- | --- | --- |
|  | Tertile 1  ≤8 (n=150) | Tertile 2  8–25 (n=151) | Tertile 3  >25 (n=147) |  |
| MACE | 38 (25.3) | 39 (25.8) | 38 (25.9) | 0.789 |
| Any revascularization | 37 (24.7) | 37 (24.5) | 36 (24.5) | 0.800 |
| Nonfatal-MI | 3 (2.0) | 5 (3.3) | 6 (4.1) | 0.750 |
| Ischemic stroke | 2 (1.3) | 5 (3.3) | 4 (2.7) | 0.647 |
| Cardiac death | 1 (0.7) | 3 (2.0) | 2 (1.4) | 0.670 |
| Variables | Tertile of Lp(a) decrease | | | p-value |
|  | Tertile 1  ≤-2.0 (n=156) | Tertile 2  -2.0–2.0 (n=149) | Tertile 3  >2.0 (n=143) |  |
| MACE | 43 (27.6) | 39 (26.2) | 33 (23.1) | 0.719 |
| Any revascularization | 41 (26.3) | 36 (24.2) | 33 (23.1) | 0.923 |
| Nonfatal-MI | 8 (5.1) | 3 (2.0) | 3 (2.1) | 0.348 |
| Ischemic stroke | 5 (3.2) | 3 (2.0) | 3 (2.1) | 0.959 |
| Cardiac death | 2 (1.3) | 0 (0) | 4 (2.8) | 0.126 |

Data are given as number (%) Lp(a): lipoprotein (a), MACE; major cardiovascular adverse event (cardiac death, non-fatal myocardial infarction, any coronary revascularization and ischemic stroke), MI; myocardial infarction
