# Supplementary material for: Comparison of apolipoprotein B/A1 ratio, TC/HDL-C, and lipoprotein (a) for predicting outcomes after PCI
Source: PLoS One. 2021 Jul 13;16(7):e0254677. doi: 10.1371/journal.pone.0254677 (PMC8277048; doi:10.1371/journal.pone.0254677)
Supplement: S4 Table — (DOCX) [file pone.0254677.s004.docx]

S4 Table. Clinical adverse events stratified by % reduction of LDL-C

| Variables | % Reduction of LDL-C≥50  (n=226) | % Reduction of LDL-C<50  (n=222) | p-value |
| --- | --- | --- | --- |
| MACE | 48 (21.2) | 67 (30.2) | 0.012 |
| Any revascularization | 46 (20.4) | 64 (28.8) | 0.018 |
| Nonfatal-MI | 8 (3.6) | 6 (2.7) | 0.484 |
| Ischemic stroke | 4 (1.8) | 7 (3.2) | 0.184 |
| Cardiac death | 1 (0.4) | 5 (2.3) | 0.078 |

Data are given as number (%) LDL-C; low-density lipoprotein cholesterol, MACE; major cardiovascular adverse event (cardiac death, non-fatal myocardial infarction, any coronary revascularization and ischemic stroke), MI; myocardial infarction
